# A Numerical Research of Herringbone Passive Mixer at Low Reynold Number Regime

**DOI:** 10.3390/mi8110325

**Published:** 2017-10-31

**Authors:** Dongyang Wang, Dechun Ba, Kun Liu, Ming Hao, Yang Gao, Zhiyong Wu, Qi Mei

**Affiliations:** 1 School of Mechanical Engineering and Automation, Northeastern University, Shenyang 110819, China; wdysend@gmail.com (D.W.); dcba@mail.neu.edu.cn (D.B.); 1500445@stu.neu.edu.cn (M.H.); 2 Silicon Steel Department, Baosteel Co., Ltd., Shanghai 201900, China; 702172@baosteel.com; 3 Research Center for Analytical Sciences, Northeastern University, Shenyang 110819, China; zywu@mail.neu.edu.cn; 4 Department of Oncology, Tongji Hospital, Tongji Medical College, Huazhong University of Science and Technology, Wuhan 4330030, China

**Keywords:** passive mixing, mixing quality, herringbone pattern, extremely low Re, electro-osmotic flow, pressure driven flow, CFD simulation

## Abstract

Passive mixing based on microfluidics has won its popularity for its unique advantage, including easier operation, more efficient mixing performance and higher access to high integrity. The time-scale and performance of mixing process are usually characterized by mixing quality, which has been remarkably improved due to the introduction of chaos theory into passive micro mixers. In this paper, we focus on the research of mixing phenomenon at extremely low Reynold number (Re) regime in a chaotic herringbone mixer. Three-dimensional (3D) modeling has been carried out using computational fluid dynamics (CFD) method, to simulate the chaos-enhanced advection diffusion process. Static mixing processes using pressure driven and electric field driven modes are investigated. Based on the simulation results, the effects of flow field and herringbone pattern are theoretically studied and compared. Both in pressure driven flow and electro-osmotic flow (EOF), the mixing performance is improved with a lower flow rate. Moreover, it is noted that with a same total flow rate, mixing performance is better in EOF than pressure driven flow, which is mainly due to the difference in flow field distribution of pressure driven flow and EOF.

## 1. Introduction

Microfluidic technique is a discipline with widespread use and rapid development [1]. With the rapid development of Lab-on-a-Chip (LOC), integrated microfluidic systems have shown great prospect due to its unique advantages, and are widely used in biochemical analysis [2,3], diagnosis detection [4], analytical chemistry [5], logical operation [6] and drug delivery [7], etc. 

As an essential component, rapid and efficient mixing acts the role of mixing two or more samples for analysis [8]. Micro mixing based on microfluidics platforms, is widely considered as though an efficient approach [9]. Conventional micro mixers are classified as active mixers and passive mixers [10,11]. In active micromixers, external energy sources, such as electrical [12], magnetic [13], and sound fields [14] are required to develop the mixing process, which makes the operation of mixing skilled-required and difficult to control. 

In passive mixers, no external energy sources are necessary. To develop mixing process efficiently, complex channel geometries and heterogeneous channel surface are utilized to increase the contact interface and decrease the diffusion distance, thus, to enhance the mixing performance [11]. With increasing need for better mixing performance, various passive mixers with novel architecture are proposed, such as butterfly mixers [15], Zigzag mixers [16], over bridged mixers [17], and herringbone mixers [18], etc. Since Stroock et al. first proposed a chaotic mixer with herringbone structure [18], passive micro mixer with herringbone pattern has become the most popular design concept due to its simple setup and robust operation. In herringbone mixers, cycled herringbone is patterned on the wall along the main channel. Based on the herringbone pattern, diffusion and advection are enhanced significantly [19,20,21]. 

To optimize the mixing performance in passive mixers, numbers of simulation work have been carried out. Computational fluid dynamics (CFD) method has been widely used to investigate mixing mechanism, yielding a better comprehension of the chaotic enhancement effects by the herringbone grooved patterns [22,23,24]. Using CFD and particle tracking methods, the effects of geometric parameters such as groove depth, number of grooves per cycle and groove width on the mixing quality. The effect of the groove asymmetry and the number of grooves per half cycle on the mixing performance has been also investigated easily, using CFD modelling coupled with Lattice-Boltzmann method [25].

In our previous experimental work, we conduct passive mixing a herringbone mixer at extremely low flow rate. Passive mixing has been developed efficiently in both pressure driven flow and electroosmotic flow. It was observed that with the same total flow rate, passive mixing driven in electroosmotic flow shows a better mixing performance. 

In this paper, we aim to study the passive mixing numerically, and novel propose a numerically study to compare the mixing under different driven modes. Specifically, we mainly focus on the effect of flow field on mixing performance in a passive mixer model with herringbone structure under different mixing modes: mixing in pressure driven flow and electroosmotic flow. Using CFD method, we conduct a numerical research on mass transfer processes inside the micro mixer. The effects of flow rate, direct current (DC) driven electric potential and herringbone structure on concentration distribution have been analyzed theoretically. Within same geometry and total flow rate, we further compare the mixing quality to disclose the reason of difference in mixing quality.

## 2. Problem Formation 

The problem originates from our previous experimental work [26]. In a herringbone micro mixer, passive mixing was developed efficiently in pressure driven flow and electroosmotic flow (EOF), respectively. It was observed that with a same total flow rate, mixing performance is better in electroosmotic flow than that in pressure driven flow.

In this work, we aim to elaborate mixing performance difference in these two driven modes theoretically, using numerical simulation. The mixing processes are simulated numerically in both pressure driven flow and electroosmotic flown with a three-dimensional (3D) herringbone structure at low *Re* regime. Based on the experimental results, we first simplify the mixer geometry to 21 mm in length, at which length a fully well-developed mixing performance was obtained experimentally. The design of the model is detailed demonstrated in Appendix A.

**Figure 1:**
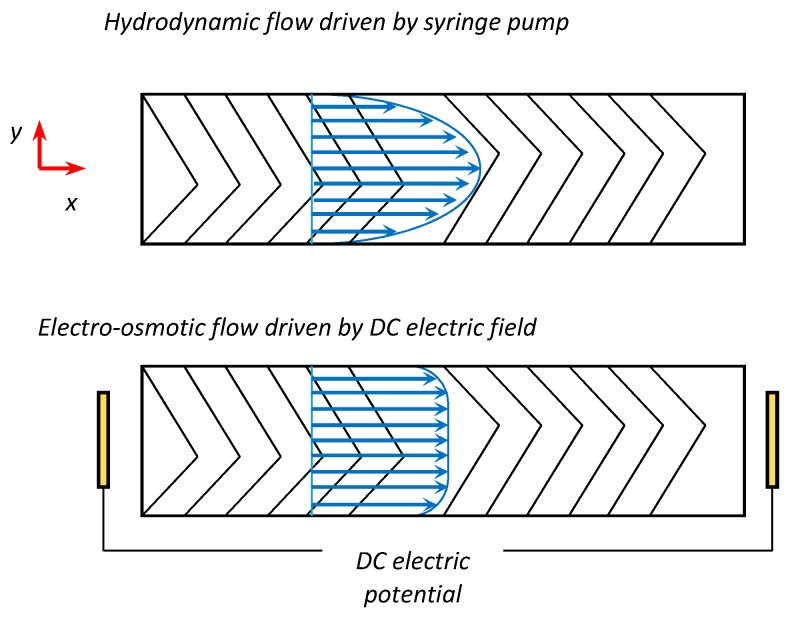
Schematic of two driven modes in this work, hydrodynamic flow and electro-osmotic flow.

Figure 1 is schematic of the physical models in this work. In a straight main channel, herringbone pattern is designed on the wall. Pressure driven flow is driven by a syringe pump at the outlet, while a DC electric field is applied to driven the electroosmotic flow. Micro mixing develops along the main channel passively.

## 3. Theory and Method

### 3.1. Pressure Driven Flow

When driven hydrodynamically by pressure, the fluid flow in the mixer is governed by continuity equation and Navier-Stokes (N-S) equation. Typical continuity equation is given by:

(1) $$\partial \rho /\partial t+\nabla \cdot \left(\rho \vec{u}\right)=0$$

While N-S equation is:

(2) $$\partial \vec{u}/\partial t+\left(\vec{u}\cdot \nabla \right)\vec{u}=-(1/\rho )\nabla P+\left(\mu /\rho \right)\nabla^{2}\vec{u}+\vec{F}$$

where ρ, $\vec{u},$ *P* and μ denote the density, velocity vector, pressure and dynamic viscosity of the liquid. Here, $\vec{F}$ represents the force exerted on the flow element at a micro scale, also known as body force. To improve simulation efficiency and obtain fine agreement between numerical calculations and experimental data, the liquid is assumed to be incompressible Newtonian fluids in CFD studies. Hence, the density of the fluid keeps constant, ∂ρ/∂t=0. Then Equations (1) and (2) can be simplified as follows:

(3) $$\mathrm{Continuity}\;\mathrm{equation}:\;\nabla \left(\vec{u}\right)\;=\;0.$$

Momentum equation (modified Navier-Stokes equation):

(4) $$\rho (D\vec{u}/Dt)=\rho \left(\partial \vec{u}/\partial t+\vec{u}\cdot \nabla \vec{u}\right)=-\nabla P+\mu \nabla^{2}\vec{u}+\vec{F}$$

### 3.2. Electro-Osmotic Flow (EOF)

The electro-osmotic flow, driven by DC electric field, is assumed to be a Newtonian fluid, and then the motion of an aqueous electrolyte solution in microchannel is governed by the N-S equations. The body force $\vec{F}$ in N-S equation is given by:

(5) $$\vec{F}=\rho_{e}\cdot \vec{E}$$

Here, $\vec{E}$ is electric field strength and $\rho_{e}$ is the density of net charge. Then N-S equation is modified into

(6) $$\rho (D\vec{u}/\;Dt)=\rho \left(\partial \vec{u}/\partial t+\vec{u}\cdot \nabla \vec{u}\right)=-\nabla P+\mu \nabla^{2}\vec{u}+\rho_{e}\cdot \vec{E}$$

Specifically, the electric field strength is given by the gradient of electrostatic potential *Ψ*,

(7) $$\vec{E}=\nabla \Psi$$

According to electric double layer (EDL) theory, the electrostatic potential Ψ and the distribution of ions in the solution by Poison Equation:

(8) $$\nabla^{2}\Psi =-\rho_{e}/\varepsilon \varepsilon_{0}$$

where *ε* is the dielectric constant of the solution, $\varepsilon_{0}$ is the permittivity of vacuum. The net charge distribution is stated by Boltzmann theory:

(9) $$\rho_{e}=-2z_{0}en_{\infty }\sin h\left(z_{0}e\Psi /k_{B}T\right)$$

Then the electrostatic potential is expressed by:

(10) $$\nabla^{2}\Psi =-(2z_{0}en_{\infty }/\varepsilon \varepsilon_{0})\sin h\left(z_{0}e\Psi /k_{B}T\right)$$

### 3.3. Mass Transfer

The species concentration in the process of diffusive mass transport is governed by the Fick’s first law (one-dimensional form):

(11) $$f=-d(\partial c/\partial x_{i})$$

Here,  c, f and d are the concentration and species flux and diffusion coefficient respectively. And $\;x_{i}\;$ denotes the diffusion direction, whereas the diffusion always happens in the opposite direction of the concentration gradient. Fick second law describes the temporal behavior of concentration profile, here in one-dimensional form.

Fick second law (one-dimensional form):

(12) $$\partial c/\partial t=d(\partial^{2}c/\partial x_{i}{}^{2})$$

Due to the presence of convection in the diffusive mixing model, an additional convective mass flow with velocity vector $\vec{u}$ extends the Fick second law to the advection-diffusion equation:

(13) $$\partial c/\partial t+\left(\vec{u}\cdot \nabla \right)c=d\nabla^{2}c.$$

### 3.4. Statistics and Evaluation

In this study, to quantify the mixing performance, a variance-based method is utilized on cut planes (*y*-*z* plane) perpendicular to the *x*-axis. In view of evaluation with statistics method, the mixing quality $\alpha_{m}$ of the mixer is described in a discrete form:

(14) $$\alpha_{m}\;=1-\sqrt{\left[\overset{N}{\underset{i=1}{\sum }}{\left(c_{i}-\bar{c}\right)}^{2}\right]/N}\;/\bar{c}$$

where $\;c_{i}\;\;\mathrm{and}\;\;\bar{c}\;$ represent different local concentration and the total mean concentration of the profile in a transfer cross-sectional plane A, which is perpendicular to the downstream direction in the main channel. The term *N* denotes the population of the sample points in the plane.

### 3.5. Simulation Method 

To properly elaborate chaotic mixing process at a micro scale, Finite Elements Method (FEM) simulations are conducted combined with a commercial software COMSOL Multiphysics 4.4 software (COMSOL Inc., Stockholm, Sweden). This work coupled computational multi-physics, including fluidics, advection diffusion transport and static electric field. Governing equations of the simulations are stated in Appendix B.

A preliminary grid dependence was conducted to minimize the influence of mesh number on the resulting mixing efficiency. Detailed mesh and time step setting are stated in Appendix C. Table 1 shows the constant parameters of the fluid used in this work. 

The boundary and initial condition is included in Table 2. For simulation of pressure driven flow, a constant flow rate, *q_w_* is exerted at outlet without any extra pressure. In the simulation of electroosmotic flow, a DC electric flied is set between the outlet and the inlet. No-slip wall condition is used in every simulation, while a zeta potential at −0.1 V is applied in the simulation of mixing in EOF. Two inlets introduce the solution and pure water into the main channel respectively. Micro mixing develops once the solution meets water at the start point of main channel. The initial concentration of the solution is 0.1 mol/m^3^ in every simulation. 

## 4. Results and Discussion

### 4.1. Mixing in Pressure Driven Flow

The first thing to realize is that flow behavior is of vital significance in passive mixing process. In this work, we aim to study the process of mixing with chaotic enhancement at the extreme low *Re* regime. Reynold number, defined as *Re* = *ρud*/*µ*, means the relative ratio of inertial forces to viscous forces. The flow rates in this work and corresponding *Re* is listed in Table 3. *Re* is this work peaks at 1.4 × 10^−2^ under a 5.0 μL/min, indicating that the whole mixing develops at an extremely low *Re* regime.

**Figure 2:**
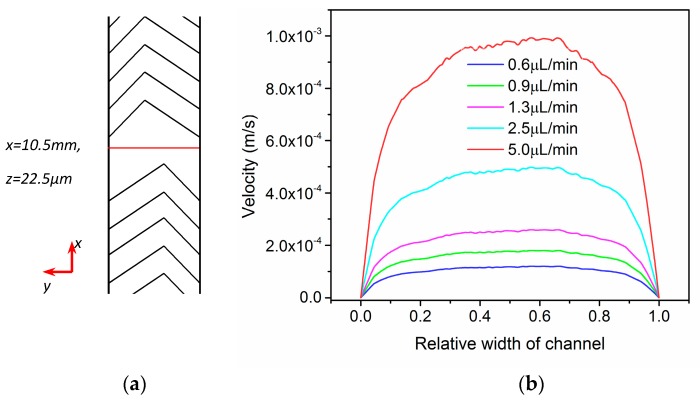
(**a**) Description of the middle position, (**b**) Velocity comparison at middle position of main channel in pressure driven flow.

To profile the flow field in the mixer, the velocity distribution across the main channel is configure and compared in Figure 2. In specific, the middle point of the main channel is taken into consideration. The red line in Figure 2a represents a series of continuous positions in *y* direction. While the *x* and *z* coordinates are 10.5 mm and 22.5 µm, which represent the middle position in both length and height direction. As can be seen, the velocity fluctuates cross the main channel in width direction, different from typical Poiseuille flow. This is mainly because of introducing of herringbone structure. The enhancing effect of herringbone structure is further investigated numerically.

**Figure 3:**
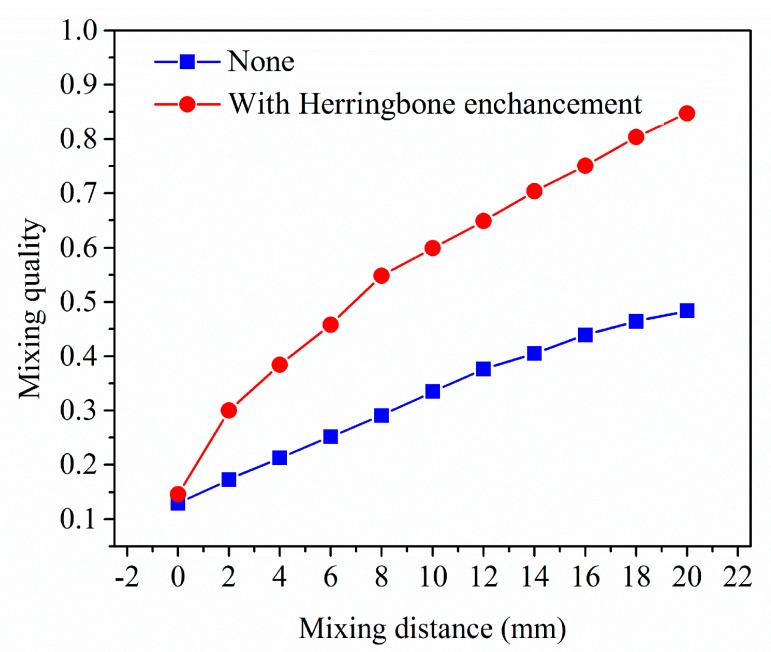
Comparison of mixing quality with and without herringbone structure. Red is the mixing quality along the main channel with herringbone enhancement, while blue is the mixing quality without any enhancement pattern.

Figure 3 compares the enhancing effect of herringbone structure on mixing quality, with a 1.3 µL/min outlet flow rate. As can been seen, the mixing performance is promoted significantly throughout the whole work.

**Figure 4:**
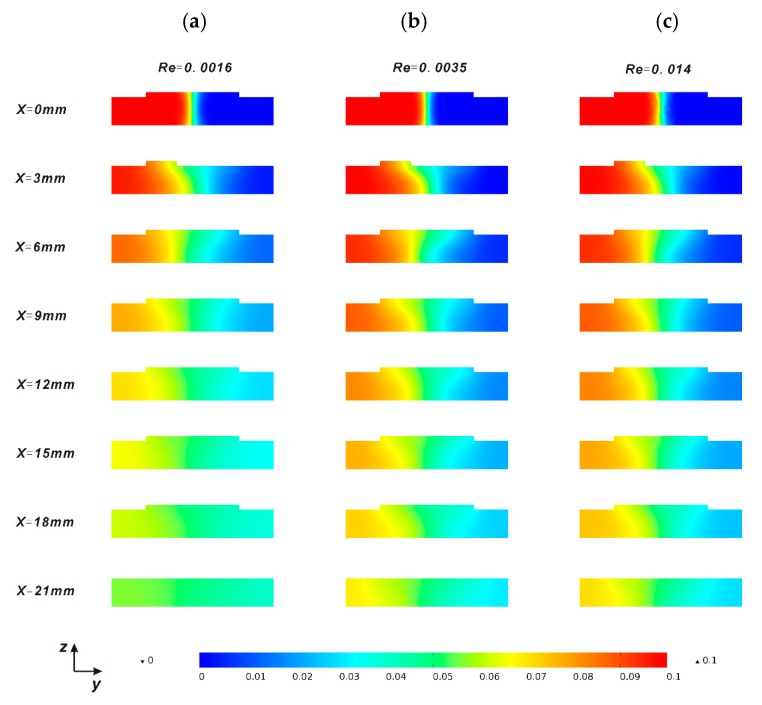
The concentration profiles along the main channel for various *Re*, (**a**) (*Re* = 0.0016); (**b**) (*Re* = 0.0035); (**c**) (*Re* = 0.014). The uniformity of the color represents the mixing performance.

The effect of outlet flow rate has been further measured, since it is directly linked to the convective flow throughout the main channel. To visualize the mixing development, the concentration profiles along the channel are demonstrated in Figure 4. At the very beginning of the mixing process (*x* = 0), the concentration distribution is same under different *Re*. As mixing develops along the channel, species distributes diversely. At outlet(*x* = 21 mm), the distribution under a lower *Re* is more uniform than that under a higher *Re*. It is concluded that when the *Re* decreases, the mixing shows a better performance. 

**Figure 5:**
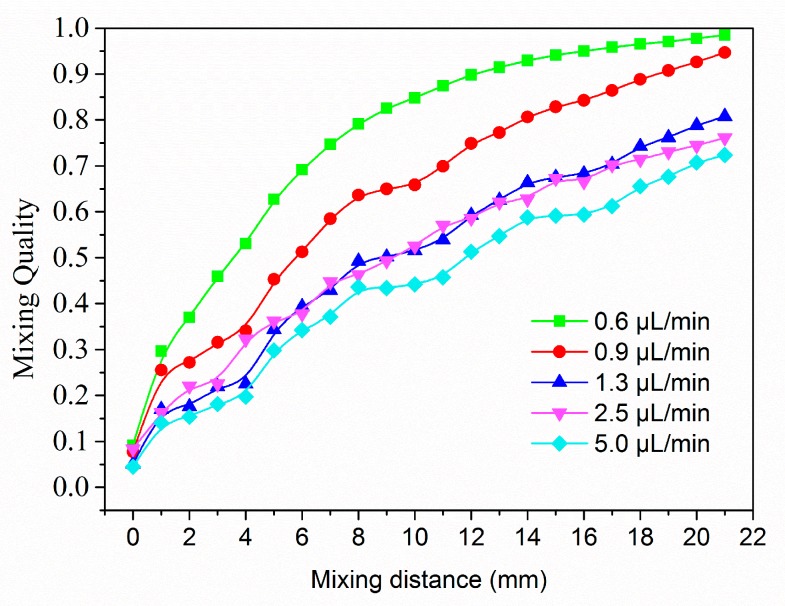
Comparison of the mixing quality along the mixing main channel under various flow rate.

Figure 5 further illustrates the effect of flow rate on mixing performance. Mixing quality along the mixing main channel for different flow rate is compared. In general, the mixing behaves better, if the *Re* goes down. The mixing quality at 20 mm increases from 72.3% to 97.7% as flow rate decreasing from 5.4 μL/min to 0.6 μL/min, which is strictly close to the results in experimental research. According to fluid dynamics, the mixing mechanism comprises convective mixing and diffusive mixing. In the main channel with herringbone patterned on the wall, the initial fluid elements are split and stretched into a multitude of smaller lamellae. Thus, a convective mixing of the species was developed. By the meantime, diffusive mass transfer occurs between the lamellae, which is enhanced by further thinning of lamellae.

The mean residence time was defined as *t_p_ = L/w*, where *L* and *w* denote the length of the channel and the mean velocity in the channel. We note that at very low Reynolds number, the mean residence time is extremely long. A decrease in *Re* leads to a decreased mean velocity and an increasing mean residence time, which in turn promotes the mixing behavior. Thus, we can conclude that in passive mixing at extremely low *Re* regime, molecular diffusion takes place and dominates the mixing processes. Lower flow field ensures a better mixing.

### 4.2. Mixing Ing Electro-Osmotic Flow (EOF)

**Figure 6:**
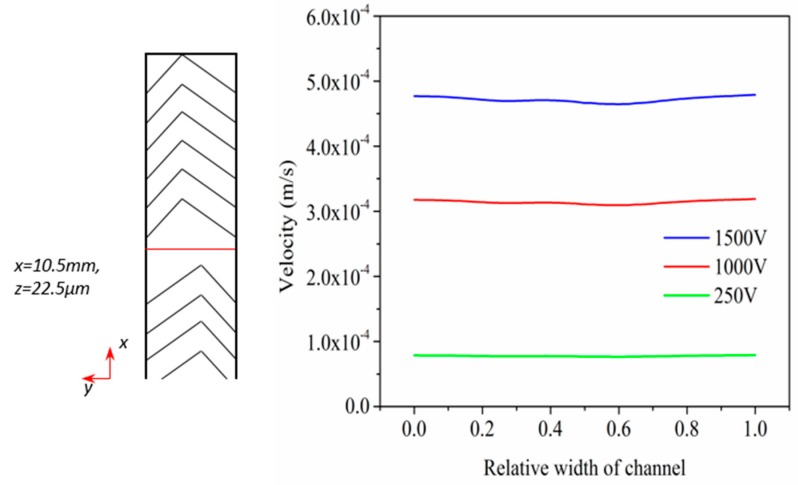
(**a**) Description of the middle position, (**b**) Velocity comparison at middle position of main channel in EOF.

In the simulation of the passive mixing in EOF, three different driven electric potential (250 V, 1000 V and 1500 V) are utilized, which is the same as the experimental setup. Similar to study of mixing in pressure driven flow above, we first investigate the flow field behavior. Flow fields are profiled using the velocity in the middle position of the channel in Figure 6, same as the continuous position utilized in the study of mixing in pressure driven flow. With a higher electric potential, the velocity increases correspondingly. However, the velocity fluctuates in y direction, indicating the disturbance of conditions herringbone structure.

**Figure 7:**
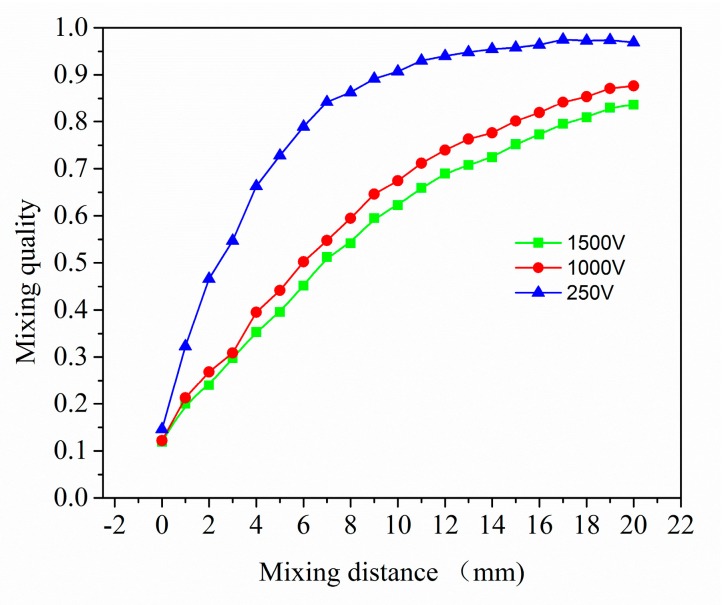
Comparison of mixing quality in Electro-osmotic flow (EOF) along the main channel with three electric potential: 250 V, 1000 V, 1500 V.

Then the effect of flow rate on mixing performance is qualified in Figure 7. It is noted that the mixing performs better under a lower electric potential, the mixing performance is improved from 0.83 to 0.97. According to the discussion above, at very low Reynolds number, the mean residence time is extremely long. A lower electric potential provides a slower flow field, and reduces *Re*. A decrease in *Re* leads to a decreased mean velocity and an increasing mean residence time, which in turn promotes the mixing behavior. Thus, we can conclude that molecular diffusion takes place and dominates the passive mixing in electroosmotic flow at extremely low *Re* regime. Lower flow field ensures a better mixing. 

### 4.3. Comparison of Two Modes

In this work, we aim to ellaborate the difference between these two driven modes and investigate the effect of flow field on mixing in both modes. In this section, the driven methods are compared in terms of flow behavior and mixing quality. 

**Figure 8:**
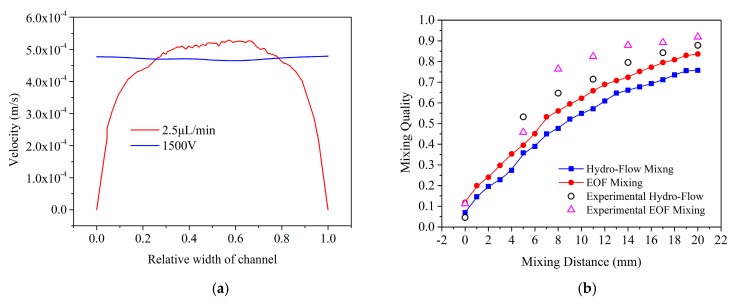
(**a**) Comparison of flow field distribution under two driven modes, the flow rate at outlet equals in both modes; (**b**) Comparison of mixing quality under both driven modes, and comparison simulation results and experimental results.

Fisrt, we compare the flow field distribution in these two modes. As shown in Figure 8a, with a same flow rate at 2.5 µL/min, the flow field distribution differs under different driven methods. As discussed above in Section 4.1 and Section 4.2, the flow fields differ from typical Poiseuille flow and electroosmotic flow, due to the influence of herringbone pattern. In comparison, in the electro-osmotic flow, the velocity shows a much lower gradient in cross direction. 

Figure 8b compares the mixing quality in both driven methods. Numerical results shows that the mixing quality improved about 4–8% under electro-osmotic driven mode compared with that of hydrodynamic flow, which is strictly close to the experimental results [23]. As discussed above, flow field is key to mixing quality. With a same geometry and total flow rate, the main factor inflencing mixing quality is local flow field distrbution. Thus, we can conclude that the main reason leading to the difference of mixing quality under these two driven modes is the distribution of flow field. 

## 5. Conclusions

In this work, we utilize CFD method to investigate the passive mixing process under two driven methods, hydrodynamic flow and electro-osmotic flow, at an extremely low *Re* regime. The mechanisms of fluid and mass transfer processes inside the micro mixer have been studied and compared. The effects of flow rate have been investigated and analyzed theoretically. Based on the simulation, we can conclude that:

1. In the simulation of passive mixing in both pressure driven flow and electroosmotic flow, the mixing quality is improved with a lower flow rate. Lower flow rate ensures a longer mean residence time. It is noted that diffusion takes the domineering factor in passive mixing in both method. While increasing the flow rate will facilitate the local diffusion, at extremely low *Re* regime, the longer residence time ensures the promotion of mixing performance.
2. Both in hydrodynamic flow and electro-osmotic flow, flow field is influenced by the herringbone structure. In hydrodynamic flow, the flow field is from typical Poiseuille flow, while in EOF the distribution of flow field is different from typical flat flow distribution in EOF.
3. The reason in the difference of mixing quality in two driven modes is the flow field distribution. With a same total flow rate, flow field distribution is totally different in pressure driven flow and electroosmotic flow. Moreover, at extremely low *Re* regime mixing performance is better in EOF than that in pressure driven flow.

## Figures and Tables

**Table 1:** Parameters of some variables utilized in this work.

Parameter	Unit	Value
ρ, density	kg/m^3^	1 × 10^3^
μ, viscosity	Pa·s	1 × 10^−3^
*ε_r_*, relative permittivity	-	8 ×10
*ε_o_*, permittivity	F/m	8.85 × 10^−12^
*d*, coefficient of diffusion	m^2^/s	1 × 10^−10^
σ, conductivity	S/m	1.0

**Table 2:** Key boundary condition and initial condition applied in the simulations of micro mixing in pressure driven flow and EOF.

Mode	Initial Condition	Boundary Condition	
Pressure driven flow	concentration	Wall condition	Outlet
	0.1 mol/m^3^	non-slip	Flow rate, *q_w_* (μL/min)
Electroosmotic flow (EOF)	concentration	Wall condition	Outlet
	0.1 mol/m^3^	non-slip, insulated, zeta	Electric potential, *E* (V)

**Table 3:** Various flow rate in this work and relevant *Re* and outlet velocity.

Flow Rate (μL/min)	0.6	0.9	1.3	2.5	5.0
Velocity (m/s)	8.5 × 10^−4^	1.28 × 10^−3^	1.85 × 10^−3^	3.56 × 10^−3^	7.12 × 10^−3^
*Re*	1.6 × 10^−3^	2.5 × 10^−3^	3.5 × 10^−3^	6.8 × 10^−3^	1.4 × 10^−2^

**Figure A1:**
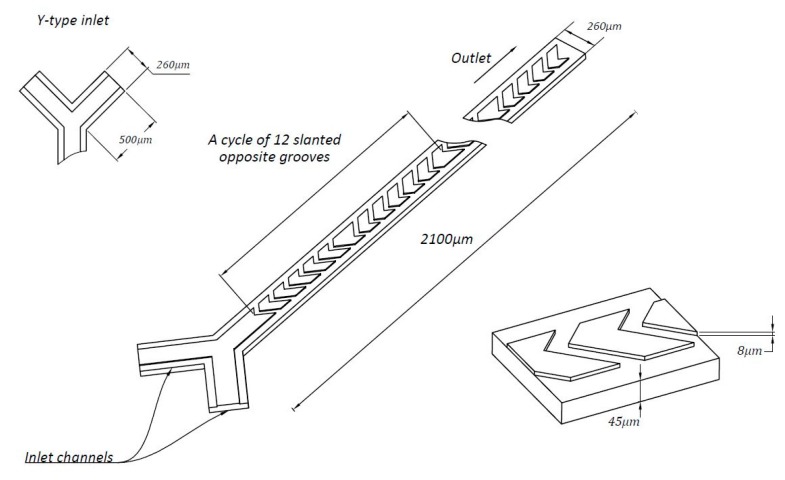
Geometry schematic of the model in micro mixing system and cycled pattern.

**Figure A2:**
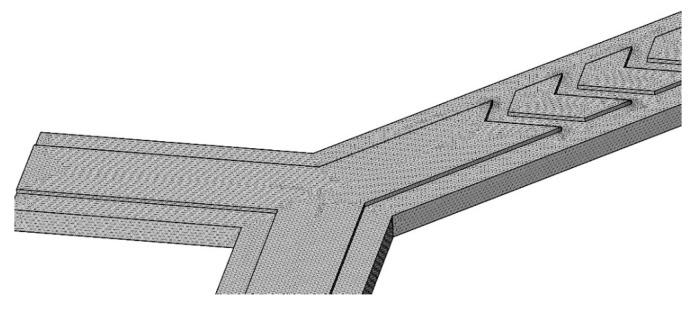
Mesh configuration of the domain at the Y-junction.

## Appendix A

The mixing system consists of a pair of Y-type inlets, a main channel and an outlet, all have a rectangular cross-section. Two incoming channels have a width of 260 μm and a length of 500 μm; the width of the main channel is 260 μm and the length is 2100 μm. The height of all the channels is 53 μm. The main channel comprises 158 slanted grooves in, forming 19 repeated herringbone structure cycles. The dimension of the grooves is 8μm in depth. Figure 1 describes the main schematic of the micro mixing system.

## Appendix B

The governing equations in simulation of mixing in pressure driven flow and EOF are as follows:

Mixing in pressure driven flow:

(A1) $$\nabla \left(\vec{u}\right)$$

(A2) $$\rho (D\vec{u}/Dt)=\rho \left(\partial \vec{u}/\partial t+\vec{u}\cdot \nabla \vec{u}\right)=-\nabla P+\mu \nabla^{2}\vec{u}+\vec{F}$$

(A3) $$\partial c/\partial t+\left(\vec{u}\cdot \nabla \right)c=d\nabla^{2}c.$$

Mixing in EOF:

(A4) $$\nabla \left(\vec{u}\right)$$

(A5) $$\rho (D\vec{u}/\;Dt)=\rho \left(\partial \vec{u}/\partial t+\vec{u}\cdot \nabla \vec{u}\right)=-\nabla P+\mu \nabla^{2}\vec{u}+\rho_{e}\cdot \vec{E}$$

(A6) $$\vec{E}=\nabla \Psi$$

(A7) $$\nabla^{2}\Psi =-\rho_{e}/\varepsilon \varepsilon_{0}$$

(A8) $$\partial c/\partial t+\left(\vec{u}\cdot \nabla \right)c=d\nabla^{2}c.$$

## Appendix C

A preliminary grid dependence has been conducted to minimize the influence of mesh number on the resulting mixing efficiency. The number of the meshes in the mesh independence testing varies from 45,165 to 5,343,381. When the element increases from 1,139,569 to 1,870,067, the resulting mixing quality at outlet shows almost no difference for every simulation input, while the mesh quality increases from 0.7369 to 0.7438. To obtain a more reliable result, the final number of elements of the mesh in this study is 1,870,067, with a 0.7438 mesh quality. Figure A2 displays the mesh grids profile. The free tetrahedral mesh is applied to the whole domain and then calibrated for fluid dynamics. The size of the mesh was customized, varying from 1.6 μm to 4 μm. Mesh modification was then performed, a curvature factor of 0.5 was used with a resolution of narrow regions remaining at 0.8.
